# Evidence triangulation in health research

**DOI:** 10.1007/s10654-024-01194-6

**Published:** 2025-03-27

**Authors:** Sirena Gutierrez, M. Maria Glymour, George Davey Smith

**Affiliations:** 1 Department of Epidemiology and Biostatistics, University of California, San Francisco, 550 16th St, San Francisco, CA USA; 2 Department of Epidemiology, Boston University School of Public Health, 715 Albany St, Boston, MA USA; 3 Medical Research Council Integrative Epidemiology Unit, University of Bristol, Bristol, UK; 4 Population Health Sciences, University of Bristol, Bristol, UK; 5 Oakfield House, Oakfield Grove, University of Bristol, Bristol, BS8 2BN UK

**Keywords:** Triangulation, Mendelian randomization, Instrumental variables, Natural experiments, Negative control studies, RCTs, Cross-context comparisons, Quantitative bias analysis

## Abstract

For many important questions about influences on clinical and public health outcomes, no single study can provide a decisive answer. The perfect study—a large, diverse, well-conducted trial randomizing all relevant versions of a treatment and comprehensively tracking all relevant health outcomes—is never feasible. Instead, we must draw conclusions by piecing together evidence from multiple imperfect studies. A systematic framework for combining disparate, complementary sources of evidence is emerging. We introduce this framework, called evidence triangulation; summarize key approaches based on delineating likely biases due to confounding, measurement, and selection; and review some methods for combining evidence. We illustrate the issues using the example of estimating the effects of alcohol use on dementia. The central tenet of evidence triangulation is to identify the most important weaknesses for any given study approach (and for each specific study applying that approach) and, if necessary, to identify which new sources of evidence that do not share these weaknesses are required. Almost certainly, the new studies will have weaknesses, but when results are consistent across studies that rest on different assumptions, and for which biases should be unrelated, the conclusions are on much sturdier ground.

## Introduction

We start by considering a public health scenario with evidence currently substantially based on epidemiological data. The health effects of alcohol consumption have been investigated statistically for over a century, but the findings remain contested. Whilst it is clear that heavy drinking has widespread adverse effects compared to abstention, low to moderate alcohol consumption is favorably associated with several health outcomes. Thus, a recent paper based on a US cohort study concluded that “low to moderate alcohol drinking was associated with better global cognition scores,” [1] and a subsequent paper from South Korea reported a similar pattern with respect to dementia onset [2]. For other health outcomes, including a range of cardiovascular conditions [3], favorable associations matching the pattern seen for dementia have been shown.

Unsurprisingly, such publications receive widespread publicity, and there is confusion about optimal drinking patterns for health. A large-scale randomized controlled trial (RCT) might allow us to adjudicate these contested interpretations, but such a trial would entail huge practical, ethical, financial, and time-scale challenges. The termination of the NIH- and industry-funded Moderate Alcohol and Cardiovascular Health (MACH) trial illustrated many of these difficulties [4]. Even if the trial had been completed, it would, at best, have answered only one narrow piece of the overall question. For example, MACH was not powered to detect effects on most cancers, would not have offered precise estimates for population subgroups, such as Asian Americans, or any estimates for people under age 50, and would not reveal consequences past the planned 10-year follow-up. Complementary study designs would be required to address these essential questions regarding the health consequences of alcohol use. In the absence of any large long-term trials, a systematic approach to sorting through the myriad study findings and integrating results from disparate study designs is required to draw conclusions. In this paper, we outline a triangulation of evidence framework that could usefully be applied to this issue and many other challenging health research questions.

## What is evidence triangulation?

Evidence triangulation is a systematic framework for evaluating causal claims based on integrating findings from diverse samples and study designs [5]. Currently, observational research on clinical outcomes is dominated by study designs that depend on controlling for observed confounders. Broadly, confounder-control approaches compare the outcomes for people observed to have different treatments and use statistical methods (e.g., multivariable regression adjustment, stratification, propensity scores, inverse probability weighting) to account for differences in the characteristics between the treatment groups [6, 7]. As such, these methods rely on having all confounders accurately measured, modeled, and adjusted for. Reverse causation—i.e., confounding by early disease processes influencing the apparent exposure—is particularly pertinent for outcomes that have a long, insidious development, such as cancer, atherosclerosis, or dementia. Causal inferences in these study designs also rest on assumptions about measurement and selection [7, 8]. The foundation for evidence triangulation is to delineate the most important assumptions necessary to answer a question of interest using each study approach. Evidence triangulation entails complementing findings from one such approach with others so that conclusions rely on different assumptions [5]. Findings can also be complemented with analyses that directly evaluate the most plausible biases—such as confounding, including through reverse causation—to strengthen inferences. Directed acyclic graphs offer convenient and intuitive representations of study designs and potential biases and can therefore be valuable for guiding evidence triangulation [9, 10] (see Box 1 for a brief introduction).

**Box 1:**
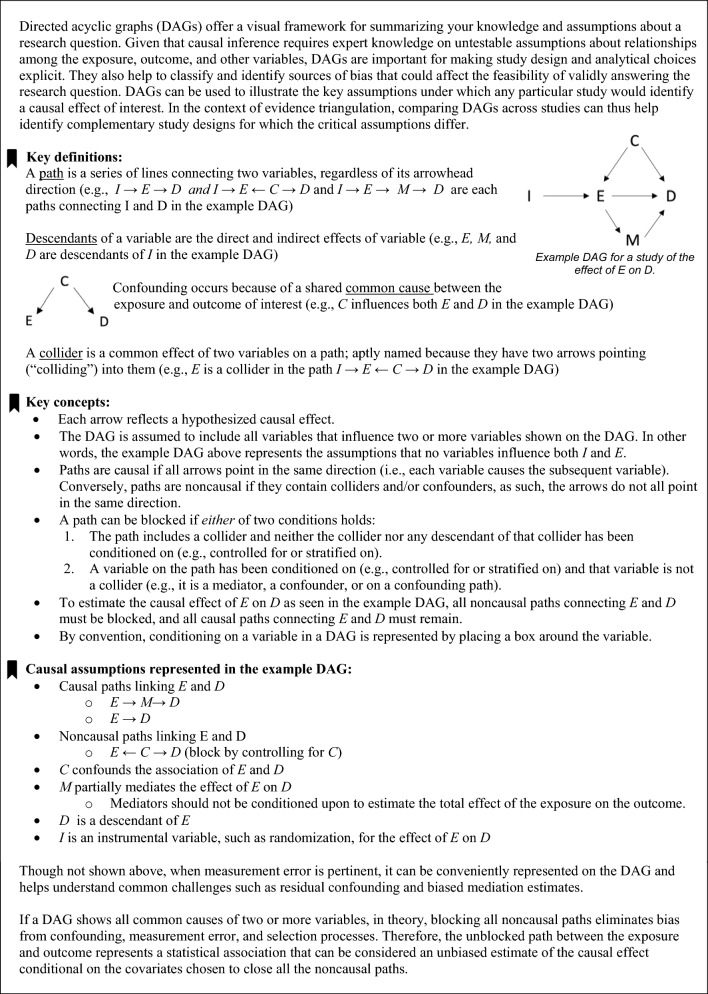
Introduction to directed acyclic graphs

For example, confounders of the association between alcohol use and dementia include both social determinants of health and clinical comorbidities. Electronic health record (EHR) data typically provide high-quality assessments of comorbidities but rarely capture detailed measures of social covariates. In contrast, survey-based studies often have high-quality measures of lifecourse social conditions but only cursory measures of comorbidities. Leveraging both EHR and survey-based data also allows for a more comprehensive assessment of varying selection processes, in addition to the differences in availability and quality of covariates. For example, in EHR-based studies of dementia risk, diagnostic bias can threaten the validity of estimates, as individuals seeking care for specific conditions are more likely to be assessed and diagnosed with dementia. To rule out selection bias, we must assume that the exposure does not influence healthcare engagement. Survey-based studies with periodic dementia assessments for all patients eliminates this concern but may face their own biases, such as individuals developing dementia and dying in between survey intervals or not participating. Through coordinated triangulation of EHR and survey-based data with the same target population, we can assess and attempt to correct for both types of confounders and selection processes, particularly if there is an overlapping sub-sample containing both sets of data.

## Specific approaches to evidence triangulation

A popular method for integrating evidence across health research studies, meta-analysis efficiently combines estimates from multiple studies with similar designs [11]. Meta-analyzed results share all of the assumptions of the contributing studies plus additional ones accompanying the statistical estimation procedure. Stratified meta-analysis and meta-regression methods better support evidence triangulation by identifying common features of studies that drive patterns of results.

Other evidence triangulation tools help synthesize results from *diverse* research designs, methods, and analytical approaches. Often bridging across disciplines and methodologies, evidence triangulation offsets the biases of each study approach, with complementary studies relying on different approaches [5]. In Box 2, we group these into methods that rely on summarizing across similar studies, methods that rely on instrumental variables instead of confounder control for causal evaluation, and tools to assess and quantify bias [6]. Each of these approaches can contribute to evaluating a causal hypothesis but depends on their own assumptions. Comprehensive descriptions of each of these exceed the scope of this paper; we recommend referring to other resources for in-depth reviews [5–7, 12–19].

**Box 2:**
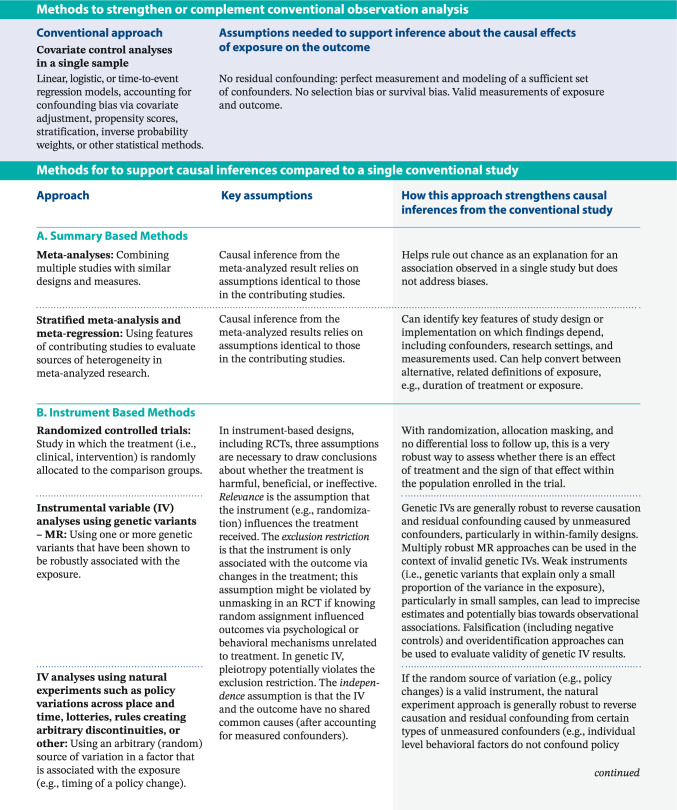

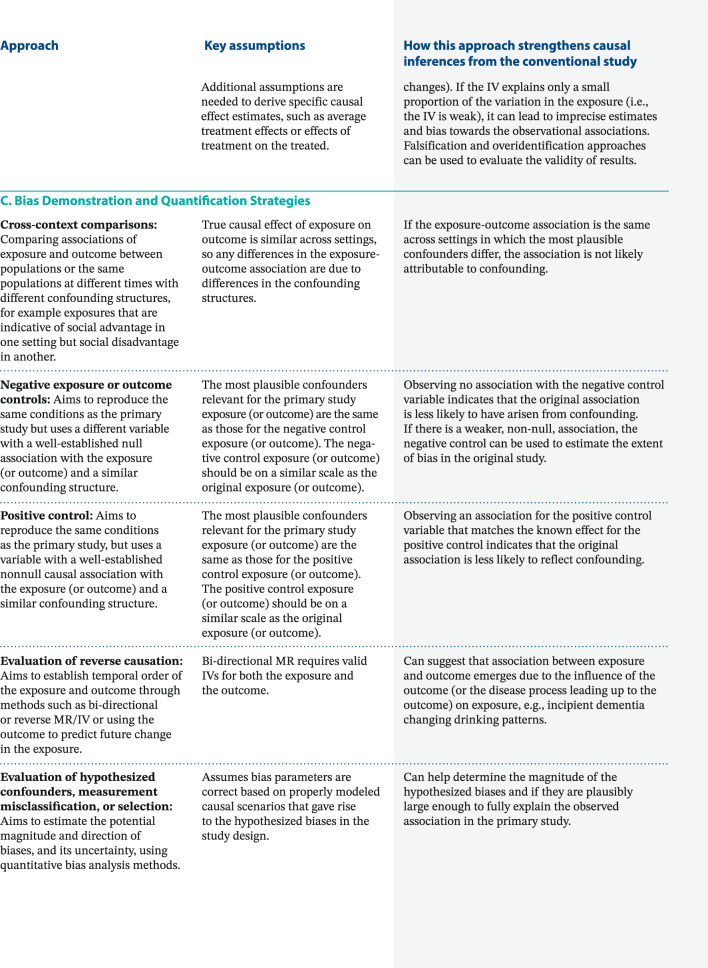
Selected approaches used for evidence triangulation:

A well-conducted randomized controlled trial (RCT) is often thought of as a powerful study design, given that randomization ensures exchangeability across groups—that is, treatment assignment is statistically unrelated to the outcomes people would potentially have under either treatment [12, 20]. However, ideal randomized trials assume full adherence to treatment, perfect blinding, and no differential loss to follow up after randomization. Thus even evidence from RCTs may be affected by selection or other biases [18]. Furthermore, the example of alcohol and dementia illustrates the limits of what can be learned from formal randomization (Box 3). Alternative instrument-based approaches leverage exogenous (arbitrary) variation in exposures such as alcohol consumption to evaluate causal effects. These designs draw on the same principles as RCTs: the process of random assignment creates a source of exogenous variation in treatment among individuals, and that source of variation in treatment is otherwise unrelated to the potential outcomes.

**Box 3:** Randomized controlled trials and evidence triangulation: idealized study designs

As our “public health scenario” we discussed how it would not be feasible to conduct an RCT that would answer all important questions about the long-term effects of alcohol on health. Because drinking alcohol across the lifecourse would likely be important, the investigator would need to randomize adolescents or young adults at the time drinking is typically initiated in the population under investigation. Effects could differ by ethnicity, gender, socioeconomic position and many other factors, so a large enough sample to examine outcomes in all combinations of these would be needed. Adequate compliance to the randomly assigned level of alcohol use would be required, and follow-up should be high. The impossibility of realizing such a comprehensive ideal study rapidly becomes apparent. This is not to claim that nothing can be learned from randomized studies—many insights are possible, and variations on conventional RCT designs may widen the applicability of RCTs to challenging exposures like alcohol use—but such studies will never deliver comprehensive evidence.	
Similarly, an idealized evidence triangulation effort can be imagined to address the same questions. A systematic review and where possible, meta-analysis of all results from each of the available study types (which might include RCTs if any have been conducted) would form the foundation of the evidence triangulation. Casting a wide net across diverse study designs—specifically seeking designs that could bolster or falsify the critical assumptions in other studies—would help in generating a more robust answer to the question. Organizing meta-analyses by the key weaknesses of the study (e.g., studies relying on no unmeasured confounders, studies relying on genetic IVs, or studies relying on policy IVs) will help reveal which assumptions are most influential to the results. Every possible violation of the assumptions required for interpreting data in relation to the question of the causal effects of alcohol on dementia would need to be considered, and energetically interrogated. A principled approach to evidence from other domains—from animal studies through to differentiated human pluripotent stem cells subjected to environmental challenges—would be required. Finally a framework for combining evidence across these domains that would allow summarizing the findings to combinable evidence would be needed. For both RCTs and evidence triangulation, we clearly rapidly enter fantasy land when thinking of the ideal study, but such an idealization can be useful for conceptualizing how more robust inference can be obtained.	

**Fig. 1:**
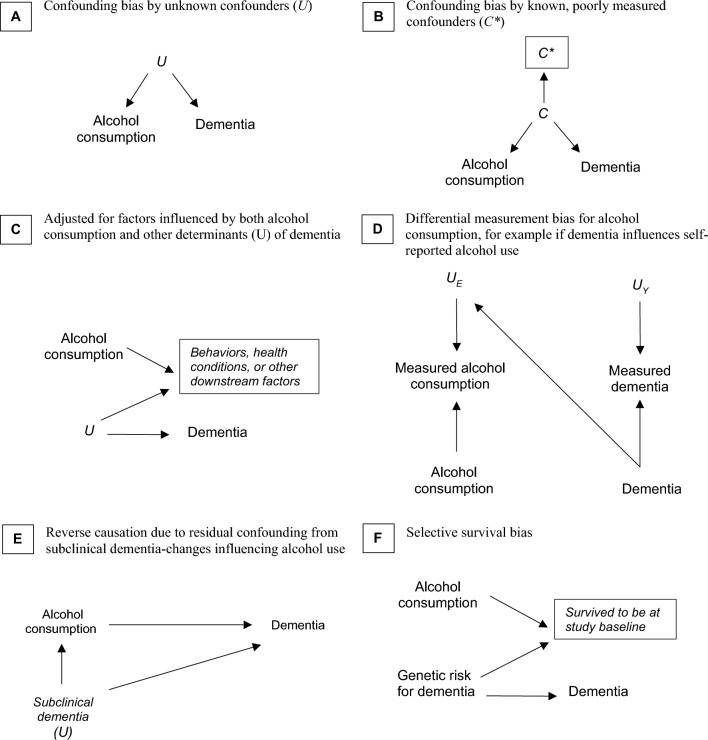
Directed Acyclic Graphs for Common Biases in the Estimated Association Between Alcohol Consumption and Dementia. The panel headings A through F correspond to the scenarios depicted in each panel. The directed acyclic graphs presented in panels A through F represent assumed data structures that could lead to biased associations between alcohol consumption and dementia. A) The direct arrows from unknown confounders *U* to alcohol consumption and to dementia indicate that they share an unmeasured common cause (e.g., lifestyle, genetics) that cannot be conditioned on; this factor leads to residual confounding bias. This is denoted by the missing box around *U* indicating a lack of statistical control for U. B) The direct arrows from known “true” confounders *C* to alcohol consumption and to dementia indicate that they share a common cause; however, C* represents the inaccurately measured value of *C*. The backdoor path from alcohol consumption to dementia via *C* cannot be fully blocked by conditioning on the measured C* leading to measurement bias. This is denoted by the box around C* indicating statistical control. C) Adjustment for downstream variables caused by alcohol consumption, such as other behaviors or health conditions, can introduce bias if the variable adjusted for is a collider influenced by the exposure and the outcome. D) Alcohol consumption is ascertained by interviewing study participants, and the presence of dementia may affect the ability to recall alcohol consumption. Therefore, one would expect an arrow from dementia to the error in the exposure measurement (*UE*). This resulting differential measurement error in the exposure can be an example of recall bias influencing the measured alcohol consumption. Non-differential measurement error (*UY*) is expected to occur if, for example, dementia cases are identified from electronic medical records, where data entry errors occur randomly and are unrelated to the true value of the exposure, alcohol consumption. As a result, one would expect arrows pointing from the true value of the outcome, dementia, and from *UY*, influencing the measured dementia. **E** Subclinical disease changes in dementia neuropathology may influence changes in alcohol consumption and will certainly be linked to subsequent dementia risk. This form of residual confounding is sometimes referred to as reverse causation, as it would appear that dementia risk *causes* changes in alcohol use. **F** The effect of alcohol consumption is harmful overall but appears beneficial at older ages because of selection or survival bias, e.g., most people who drink alcohol who are susceptible to developing dementia due to their alcohol consumption and genetic risk of dementia do so by a specific age threshold, and thus this age group of older adults without dementia at study baseline is depleted of participants who drink alcohol and would be susceptible to dementia due to their genetic risk

Changes in policies and genetic variation (to name two) can be used as instrumental variables (IVs) to circumvent confounding and other biases [13, 15, 19, 21]. The strengths of these instrument-based designs are that they can quantify how this variation in treatment induced by a valid instrument affects the outcome despite the presence of unmeasured confounders of the treatment-outcome association (see Fig. 1 for DAGs illustrating assumptions). Changes in policies, especially if the changes were introduced with essentially arbitrary timing, have long been used for IV analyses in economics research [22]. The relatively recent investment and availability of human genomic data [23] gave rise to the formalization of Mendelian Randomization (MR) approaches [15, 24], which use genetic instruments (e.g., single nucleotide polymorphisms) and leverage the natural assortment of genetic variants to explore the causal effects of modifiable risk factors [19]. Since its early applications, numerous extensions of MR methods have allowed for estimates that are more robust to possible violations of assumptions and address more flexible substantive questions such as effects at different stages of the lifecourse and non-linear dose–response effects [15, 19, 25–29].

However, these instrument-based study designs come with their own set of assumptions. For the purposes of evidence triangulation, the key strength is that these assumptions are not the same as for conventional confounder-control methods [13, 21]. Even if there is unmeasured confounding of the exposure and outcome, IV-based methods can identify causal effects with additional assumptions being required to understand the generality of this effect estimate within a population [15, 17, 19]. The most critical assumption in IV methods is usually that the IV has no reason to be associated with the outcome other than through its association with the exposure. If the IV is unrelated to the outcome, it implies that the exposure has no effect on the outcome. If the IV is related to the outcome, the magnitude of this association can be used to estimate the effect of the exposure on the outcome. The assumptions for IV analyses are identical to the assumptions for RCTs; for example, in an RCT, we assume that randomization is only associated with the outcome via the treatment received [6]. Randomization can be conceptualized as an ideal IV, and the (often considerable) degree to which an IV-based design deviates from the investigator-randomized ideal is an indicator of possible biases in an IV-based study. However, IV methods have less statistical power than conventional analyses of the same question obtained from the same sample. Thus, evaluating the confidence interval for IV estimates is essential to guide interpretation; in many cases, little is learned from an IV analysis because the instrument is weakly tied to the exposure of interest or the sample size is too small.

Several evidence triangulation approaches can be applied to uncover or circumvent residual confounding, including comparisons of outcomes within sibships (e.g., sibling controls, discordant sibships, twin studies) [19, 30, 31], in different populations or contexts (e.g., high- versus low-income countries) [32], or using negative controls [14, 16, 17, 19, 28, 33]. Comparisons between siblings with discordant exposures avoid confounding by factors shared between siblings, even if these factors are unmeasured [30, 31]. Cross-context comparisons leverage multiple populations where the confounders of concern in one population are either irrelevant or are expected to induce an opposite direction of bias in another population or context [32]. If the association observed in the original population is causal, we expect the association to persist across different populations or contexts, regardless of the different nature of the confounding structure. If the association observed in the original population is not causal, we expect the association to vary across different populations or contexts with different confounding or selection biases. If the association is consistent across settings, it supports causal inference. This inference is based on the assumptions that the causal effect is similar across the different settings (i.e., external validity), an assumption that must be considered on a case-by-case basis if the estimates differ across settings. Additionally, leveraging different samples with varying selection processes can help shed light on the influence of selection bias, which occurs if there is any deviation between the target estimand and the expected value of the estimate in the samples [34]. Selective enrollment may lead to estimates that do not accurately reflect the true causal effect for the target population you intend to extend your findings to, driven by an unequal distribution of effect modifiers between the study sample and target population. One example is the use of differential recruitment strategies in dementia research, such as those employed by memory clinics versus community-based approaches [35]. These differing selection processes lead to varying distribution of sociodemographic characteristics of participants which in turn may introduce bias (e.g., collider stratification).

Negative controls are exposures or outcomes for which the confounding structure is likely similar to that in the original study but for which causal effects are implausible [36]. For example, paternal smoking has been used as a negative control exposure when evaluating the intrauterine impact of maternal smoking on offspring obesity [37]. Maternal and paternal smoking generally have similar confounding structures with offspring outcomes, but intrauterine mechanisms influencing offspring obesity should be minimally affected by paternal smoking. Thus, the association of paternal smoking and offspring obesity will detect the presence of residual confounding in the association between maternal smoking and offspring obesity. Negative controls can detect the presence of residual confounding, recall bias, and other analytic flaws, including reverse causation and selection bias [8, 16, 28]. Positive controls are used in the context of a well-established non-null causal association with the exposure or outcome, which should be identified by any reliable method being used for effect identification or estimation.

Box 2 summarizes the above and additional methods, including using IV methods to evaluate reverse causation and quantitative bias analyses [14]. In dementia research, concerns such as selective survival, differential loss to follow up, and reverse causation are particularly pernicious, as they may be inherent to the study design. Participants need to survive to old age, and preclinical disease changes may influence the exposure of interest [38]. Strategies for detecting and quantifying bias, can help correct estimates [14, 39, 40]. For example, if the selection process is known or can be modeled, the parameters can be used to correct for selection bias. Results from these sensitivity analyses can demonstrate the robustness of findings—showing whether results remain consistent under the hypothesized selection processes. IV methods can be used to account for selection bias due to censoring, provided a valid instrument exists to account for unmeasured predictors of such censoring [41].

Lastly, supportive evidence from other sources, such as laboratory experiment data, might provide a theoretical foundation or help explain the mechanisms underlying the phenomena in question. Other data sources (e.g., temporal trends) could provide real-world context, as indeed could perspectives or perspectives from participants. One caveat is that stories are deceptively easy to weave from preliminary or ambiguous findings. Our own confirmation biases [42] can affect the interpretation of our results. As such, evidence triangulation can serve as a ‘checks and balance’ system to objectively evaluate evidence and help overcome confirmation bias, other external pressures, and conflicts of interest. The more comprehensively we can explore multiple lines of evidence, the more convincing the overall argument becomes. Just as benchmarking against an ideal, hypothetical RCT can help strengthen observational studies, benchmarking against an ideal evidence triangulation approach may be useful (Box 3).

## Quantitative versus qualitative triangulation

**Fig. 2:**
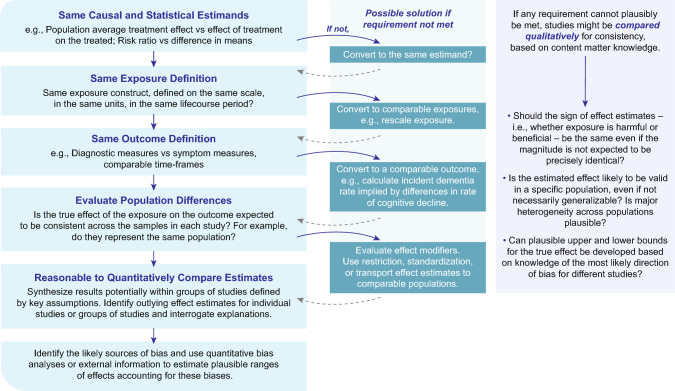
Schematic for how to assess comparability of estimates when contrasting or combining results from different studies

Triangulation requires comparing estimates from different studies, but inconsistencies in populations, measurement instruments, study design, or analytic procedures may render quantitative syntheses impossible. It is necessary to initially evaluate whether formal quantitative triangulation can be completed or whether the interpretation of findings from different studies must be largely qualitative (Fig. 2). Both content understanding and statistical tools are needed to understand whether direct comparisons are appropriate and how to correct or modify estimates from different studies to allow comparisons. One of the first considerations is whether the studies address the same target question. This requires evaluating the consistency of estimands, exposure definitions, and outcome measures across studies. For example, studies of essentially the same research question may focus on different causal estimands (e.g., the effect in the entire population versus the effect in a subgroup) or on different statistical estimands (e.g., the odds ratio versus a risk ratio). It is important to assess whether different causal or statistical estimands would be expected to substantively diverge. Even when statistical estimands differ, the estimates can often be converted or transformed to make quantitative triangulation feasible. As an example of scaling of estimands we can consider triangulation of the well-established causal effect of LDL cholesterol (LDLc) on coronary heart disease (CHD) risk. In RCTs the estimand is the effect of lowering LDLc for the duration of the trial, say 5 years. In MR, the estimand relates to the influence of the genetic variants on the trajectory of LDLc from birth (or before) onwards [43, 44]. Given the cumulative nature of atherosclerosis development, this will yield substantially greater magnitude of effects for a given difference in LDLc than that seen in RCTs. The scaling of these—roughly 40% of the effect seen in RCTs compared to MR studies—is relatively stable, and in principle would allow combination of findings between the two study designs [45, 46]. Importantly if two independent estimates can be generated from the same study these can legitimately be combined using the “evidence factors” framework [47].

Exposure definitions must also be evaluated for consistency across studies, as different measurements or operationalizations of similar exposures may be used. In some cases, these differences may be resolved with a simple transformation —alcoholic drinks per day can be rescaled to drinks per week. In other instances, additional information is necessary to harmonize the measures —binge drinking defined as 4 + drinks in an episode versus 5 + drinks in an episode—or the differences may be irreconcilable because the measured constructs are distinct—binge drinking frequency versus average daily drinks. One example of this challenge arises when comparing estimates from MR to estimates from conventional observational studies. MR studies nearly always estimate effects of an unknown series of alterations in the exposures induced by the genetic instruments across the life course [44, 48]. Although genetic variants often have time-varying effects, too little is known about age-specific genetic of most phenotypes to incorporate this into a quantitative comparisons with observational studies. Similar ambiguity about the exposure duration applies to many observational studies, with the exception of “new-user designs”. In “new-user designs”, the study population is restricted to individuals who are initiating the treatment for the first time, which helps reduce biases related to prior treatment history and more accurately captures the effects of the treatment from the outset. If exposure is simply coded as present or absent at the beginning of follow-up, it may correspond with very long duration of past exposure. For example, individuals who report heavy drinking at study enrollment likely have a past history of heavy drinking, which is rarely quantified or incorporated into the exposure assessment. Similarly, BMI at any given age is correlated with earlier life BMI, and thus conventional observational studies evaluating the links between BMI measured at a single time point and subsequent health will conflate lifecourse BMI trajectories with baseline BMI. In some situations with robust evidence regarding age-specific genetic instruments and a clear biological rationale—e.g., comparing pre-pubertal BMI with middle-age BMI and using positive and negative controls—meaningful MR analysis may be possible [29]. Similarly, outcome definitions may vary across studies. For instance, in dementia research, the same underlying pathologic processes may influence all-cause dementia, clinically diagnosed dementia, mild cognitive impairment, cognitive decline on a global mental status measure, and memory loss. While studies using different outcome assessments can sometimes be reconciled statistically, this is not always the case.

Next, it is crucial to evaluate the external validity of the studies. The comparison of studies requires considering the population samples they include and evaluating the presence of heterogeneity. Comparing estimates across populations with different confounding structures is a valuable evidence triangulation approach to reveal bias, but interpreting such comparisons requires asking whether the true causal effect might differ across the populations. Direct comparisons are appropriate if the samples from each study represent the same population, but often the populations represented differ. More commonly, when studies are recruited from clinical care settings, the samples are not representative of *any* clearly defined population. In this setting or when the question relates to prognosis or outcomes of a clinically diagnosed condition, the potential role of selection bias or heterogeneity of effects between samples must be considered. For example, the effect of alcohol consumption in a sample of people with hypertension or a group of people with a family history of dementia might differ from the effects in a general community-based sample. The collider bias inherent in selecting a sample of such individuals who suffer a particular medical condition may generate additional bias of the observed association away from the causal effect. This collider bias may masquerade as effect heterogeneity [10, 15, 17–19]. Statistical methods, such as restriction or standardization, may be applicable to adjust or transport effect estimates across different study populations. Finally, it is important to assess the feasibility of quantitatively combining the estimates, while adjusting for identified biases. Identifying sources of bias—both in terms of direction and magnitude—for each study approach will help determine the feasibility of quantification. A thorough examination of potential sources of heterogeneity between study approaches will often provide more valuable insights than attempting to calculate a single overall effect measure. Even when estimating a single overall effect does not make sense given heterogeneity across the estimands, measures, or populations, then quantitative comparison may still be informative. Noting whether estimates differ in sign or differ radically in magnitude can guide interpretation. The connection between evidence and decision-making is coarse: the quality of evidence relevant for informing clinical, behavioral, or policy decisions is sometimes as crude as whether there is a beneficial or harmful effect.

The methodology of quantitative triangulation of evidence is very much a work in progress [47, 49], with the development and testing of different approaches. Even when quantitative comparisons are not appropriate, qualitative comparisons across multiple studies relying on different assumptions can offer substantial insight, certainly superior to that from use of a single study approach. In some cases, studies would be expected to deliver estimates of the same sign or estimates of similar magnitude if both were unbiased, even if they would not necessarily provide *identical* point estimates. A specific subgroup in which the effect estimate is likely to be valid may be identified, and with subject matter knowledge being required to evaluate if the estimate can reasonably be generalized. When the likely direction of bias in different studies is clear, comparing results across studies may indicate upper and lower bounds for the true effect: i.e., the true effect size likely falls between estimates from upwardly biased and downwardly biased studies. When results from different approaches lead to qualitatively different conclusions, our confidence in causal inferences from any study must be tempered. When results vary across studies, explicitly noting the sources and directionality of the biases can help prioritize future studies that are needed to move towards a robust causal answer or indicate if a conclusion might be reached with the current body of research. In Box 4, we provide an example of qualitative triangulation of evidence for the relationship of alcohol and dementia, leveraging approaches with different biases [50–58].

**Box 4:** Triangulation of evidence: the case of alcohol use and dementia risk

Alcohol use is a controversial risk factor for dementia [50]. As with its association with other health outcomes, the causality has been widely debated, particularly with respect to the dose and frequency of alcohol consumed. Much of this debate stems from earlier research studies suggesting a potential beneficial effect of light-to-moderate alcohol consumption (compared to no alcohol consumption) on cardiovascular disease, a known risk factor for dementia [51, 52]. However, alcohol consumption–especially in the United States–is influenced by socioeconomic status, regional norms, religious beliefs, and past substance use experiences such as addiction, each of which may independently influence dementia risk [53]. These strong confounders complicate the interpretation of observational findings (see Fig. 1 for a graphical depiction). Additionally, measurement and selection biases are common challenges to contend with in research involving older populations, superimposing additional biases in typical study designs. The controversy persisted due to the inherent difficulties in studying the long-term effects of alcohol on health, as illustrated by the termination of the Moderate Alcohol and Cardiovascular Health (MACH) trial. The feasibility of conducting randomized controlled trials (RCTs) is limited given the varying lengths of a cumulative exposure to alcohol over a prolonged preclinical period. Below, we review several studies employing different methodological approaches to help illustrate how a more robust evidence base can be built to address the decades-long debate surrounding the potential cognitive health benefits of light-to-moderate alcohol consumption.	

Approach	Source of bias and direction
A. Conventional covariate control	A key source of bias in this approach is residual confounding. Many of the studies included adjusted for age, sex, education, but did not account for other potential confounders, such as income, occupation, health behaviors, alcohol use prior to study enrollment, morbidities and their time-varying nature. This limitation could result in an effect estimate that is an **exaggeration** of any real protective effect. In addition, many of these studies did not use statistical approaches to account for **differential selection into the study**, given the competing risk of death for alcohol and dementia-related mortality.
Systematic review and meta-analysis [54] of prospective cohort studies, primarily involving participants of European or U.S. origin (n = 73,330), found that no to low alcohol consumption (≤ 12.5 g/day) was associated with the lowest risk of dementia, with a nonlinear relationship observed.	
B. Twin studies	Diagnosis of dementia was assessed through death registries or by telephone interviews, potentially misclassifying the outcome, which may **attenuate** estimated effects. Because the number of twins discordant for alcohol use is small, estimates are imprecise. Ascertainment of alcohol consumption may also suffer from misclassification. However, twin studies help account for genetic and environmental confounding.
A Swedish twin birth cohort (1907–1925) [55], found no significant association of low (1–5 g/day) or moderate (6–12 g/day) alcohol consumption with dementia compared to abstention. A Finnish twin cohort (ages 65 +) [56], also found the lowest risk of dementia among individuals with low-dose alcohol consumption (> 0 to < = 5 g/day).	
C. Mendelian randomization (MR) studies	Assuming no pleotropic effects of the genetic variants that influence the alcohol consumption and dementia risk, MR helps account for residual confounding and reverse causation. Methods for testing and potentially controlling for pleiotropy, as well as the use of a positive control, were used to help address potential biases. Non-linear MR may have limited statistical power to detect non-linearities. Inconsistent results between overall incidence of Alzheimer’s versus age of onset suggest either strong selective survival biases, other types of pleiotropic associations, or chance findings.
Two MR studies [57, 58] used genetic variants identified to influence alcohol consumption in genome-wide association studies of European participants. Using these genetic variants as instrumental variables, one study found evidence of positive linear associations of alcohol consumption with Alzheimer’s disease risk and the other found no association with Alzheimer’s disease risk but did find evidence that alcohol consumption led to an earlier age of onset of Alzheimer’s disease. Although conventional analyses in the data indicated a non-linear association between alcohol consumption and Alzheimer’s risk, non-linear MR analysis did not detect a J-shaped association among current drinkers [58].	

## Remaining challenges

Evidence triangulation takes advantage of studies using many different approaches, and for each approach, a wide range of analyses could be carried out. This introduces the possibility that researchers could conduct many analyses and cherry-pick the findings that most closely match their expectations. P hacking and unacknowledged hypothesizing after the results are known (HARKing) are major problems in biomedical research, as in other empirical fields, Vibration of effects analyses–systematically iterating analyses across a wide range of plausible alternative specifications—is a potential antidote to researchers differentially pursuing analytic decisions that confirm their expectations [59]. More overt forms of cherry-picking by investigators include analyzing several potential negative control outcomes and reporting only the one that fits best with their hypothesis. This possibility is exacerbated by publication bias if editors or reviewers take a dim view of unexpected and null results. Pre-registration of a protocol for a triangulation exercise before analyses are conducted would, in principle, circumvent this possibility [60]. The balance of advantages with potential downsides of pre-registration in the triangulation field are not well characterized at present [61], meriting formal evaluation of this practice.

Integrating evidence from completely unrelated study designs remains a challenge. Our discussion above emphasizes qualitative comparisons when quantitative comparisons are not feasible. Additional methods and substantive guidance to more formally combine findings from heterogeneous sources are needed. Although in this discussion, we have not focused on measurement triangulation—i.e., tools to crosswalk between related but somewhat different measurement instruments—this is an essential step in evidence triangulation [62, 63], as is clear in Fig. 2. The specific tools used to measure exposures, outcomes, modifiers, or covariates differ across studies, and it is a foundational step to calibrating different measures of the same constructs against one another. These may include any number of approaches, such as psychometric methods or measurement crosswalks developed by randomizing people to different measures of the same construct.

Health research data sets have historically originated predominantly from high-income countries and overrepresent socially advantaged individuals who are more likely to participate in clinical or cohort studies. In the context of studies of older adults, recent work has expanded to include cohorts from low- and middle-income country settings, but data opportunities to support cross-context comparisons remain a high priority. Relatedly, methods based on IVs generally require large samples to derive precise estimates. For policy-based IVs, the samples require geographic heterogeneity across settings with different policies. Thus, large data sets, often from surveillance systems such as vital statistics sources, are essential. Deriving high-quality measures of outcomes of interest from these sources and creating opportunities to link to IVs (including genetic measures) is an important challenge. Most importantly, increasing the accessibility of data is a crucial step forward in strengthening the reliability of published research in general, and triangulation in particular [64].

Finally, we see tremendous opportunity to honestly report the magnitude of biases in real data. Estimation of the potential quantitative influence of biases given reasonable assumptions should be applied whenever feasible. Many theoretically plausible biases turn out to be relatively minor in applied data settings, but in some situations it is clear that biases produce strong and consistently misleading findings [65–67]. Evidence on which confounding or selection processes are major sources of bias for specific research questions could enhance the validity of conclusions from studies even when those threats cannot be directly quantified, through quantitative bias analysis methods. If, however, all presumed biases consistently operate in the same direction and with similar magnitudes across various study designs, triangulation may not necessarily increase confidence in the robustness of the findings. There is also a possibility that different approaches may inadvertently share the same biases in both direction and magnitude. To mitigate this limitation, it is crucial to move beyond studies that rely solely on data generated from similarly biased sources. Instead, robust triangulation requires incorporating a variety of evidence drawn from different methodologies and domains of data, which helps to reduce the likelihood of systematic bias and provides a more comprehensive understanding of the research findings. The strengths and limitations of the variety of evidence thesis are discussed formally elsewhere [68, 69].

## Conclusion

A common concern about evidence triangulation is that such an approach will uncover clear divergencies. Perhaps we ought not look under the rock because we may find a nest of troublesome snakes? This head-in-the-sand approach is antithetical to science. To the contrary, the most interesting moment in science is when unexpected findings emerge. These are moments with the most potential to alter our understanding of the mechanisms of disease and make meaningful advances. In the words of William Bateson, “Treasure your exceptions.” [70]. Triangulation of evidence is a principled way to identify such treasures.

Formal quantitative approaches to evidence triangulation are a relatively recent innovation in health research. These methods are sparking interest because they align with long-standing scientific intuitions and have the potential to address key challenges in clinical and population health research [71, 72]. Evidence triangulation can improve confidence in our results, considerably extend the implications of RCTs, avoid unfounded conclusions, identify settings with truly divergent findings, kindle meaningful new insights when incongruities are recognized, and stimulate new research with the highest chance of being truly informative.

## References

[1] Zhang R, Shen L, Miles T, et al. Association of low to moderate alcohol drinking with cognitive functions from middle to older age among US adults. JAMA Netw Open. 2020;3(6): e207922. 10.1001/jamanetworkopen.2020.7922. 32597992 10.1001/jamanetworkopen.2020.7922 PMC7324954

[2] Jeon KH, Han K, Jeong SM, et al. Changes in alcohol consumption and risk of dementia in a nationwide cohort in South Korea. JAMA Netw Open. 2023;6(2): e2254771. 10.1001/jamanetworkopen.2022.54771. 36745453 10.1001/jamanetworkopen.2022.54771 PMC12549098

[3] Bell S, Daskalopoulou M, Rapsomaniki E, et al. Association between clinically recorded alcohol consumption and initial presentation of 12 cardiovascular diseases: population based cohort study using linked health records. BMJ. 2017;356: j909. 10.1136/bmj.j909. 28331015 10.1136/bmj.j909 PMC5594422

[4] Mitchell G, Lesch M, McCambridge J. Alcohol industry involvement in the moderate alcohol and cardiovascular health trial. Am J Public Health. 2020;110(4):485–8. 10.2105/AJPH.2019.305508. 32078349 10.2105/AJPH.2019.305508 PMC7067094

[5] Lawlor DA, Tilling K, Davey SG. Triangulation in aetiological epidemiology. Int J Epidemiol. 2016;45(6):1866–86. 10.1093/ije/dyw314. 28108528 10.1093/ije/dyw314 PMC5841843

[6] Matthay EC, Hagan E, Gottlieb LM, et al. Alternative causal inference methods in population health research: evaluating tradeoffs and triangulating evidence. SSM - Popul Health. 2020;10: 100526. 10.1016/j.ssmph.2019.100526. 31890846 10.1016/j.ssmph.2019.100526 PMC6926350

[7] Lash TL, VanderWeele TJ, Haneuse S, Rothman KJ. Modern epidemiology. 4th ed. Wolters Kluwer/Lippincott Williams & Wilkins; 2021.

[8] Lawlor DA, Davey Smith G, Kundu D, Bruckdorfer KR, Ebrahim S. Those confounded vitamins: What can we learn from the differences between observational versus randomised trial evidence? Lancet. 2004;363(9422):1724–7. 10.1016/S0140-6736(04)16260-0. 15158637 10.1016/S0140-6736(04)16260-0

[9] Digitale JC, Martin JN, Glymour MM. Tutorial on directed acyclic graphs. J Clin Epidemiol. 2022;142:264–7. 10.1016/j.jclinepi.2021.08.001. 34371103 10.1016/j.jclinepi.2021.08.001 PMC8821727

[10] Greenland S, Pearl J, Robins JM. Causal diagrams for epidemiologic research. Epidemiology. 1999;10(1):37–48. 9888278

[11] Egger M, Higgins JPT, Davey Smith G (eds) *Systematic Reviews in Health Research: Meta-Analysis in Context*. Wiley; 2022. 10.1002/9781119099369.ch1

[12] Deaton A, Cartwright N. Understanding and misunderstanding randomized controlled trials. Soc Sci Med. 1982;2018(210):2–21. 10.1016/j.socscimed.2017.12.005. 10.1016/j.socscimed.2017.12.005 PMC6019115 29331519

[13] Greenland S. An introduction to instrumental variables for epidemiologists. Int J Epidemiol. 2000;29(4):722–9. 10.1093/ije/29.4.722. 10922351 10.1093/ije/29.4.722

[14] Lash TL, Fox MP, MacLehose RF, Maldonado G, McCandless LC, Greenland S. Good practices for quantitative bias analysis. Int J Epidemiol. 2014;43(6):1969–85. 10.1093/ije/dyu149. 25080530 10.1093/ije/dyu149

[15] Sanderson E, Glymour MM, Holmes MV, et al. Mendelian randomization. Nat Rev Methods Primer. 2022;2:6. 10.1038/s43586-021-00092-5. 10.1038/s43586-021-00092-5 PMC7614635 37325194

[16] Lipsitch M, Tchetgen ET, Cohen T. Negative controls: a tool for detecting confounding and bias in observational studies. Epidemiology. 2010;21(3):383–8. 10.1097/EDE.0b013e3181d61eeb. 20335814 10.1097/EDE.0b013e3181d61eeb PMC3053408

[17] Hernán MA, Robins JM. Causal inference: what if. Chapman & Hall/CRC Press; 2024.

[18] Hernán MA, Hernández-Díaz S, Robins JM. A structural approach to selection bias. Epidemiology. 2004;15(5):615–25. 10.1097/01.ede.0000135174.63482.43. 15308962 10.1097/01.ede.0000135174.63482.43

[19] Davey Smith G, Richmond R, Pingault JB (eds) *Combining Human Genetics and Causal Inference to Understand Human Disease and Development*. Cold Spring Harbor Laboratory Press; 2022.

[20] Cook TD. Twenty-six assumptions that have to be met if single random assignment experiments are to warrant “gold standard” status: a commentary on Deaton and Cartwright. Soc Sci Med. 1982;2018(210):37–40. 10.1016/j.socscimed.2018.04.031. 10.1016/j.socscimed.2018.04.031 29778288

[21] Angrist JD, Imbens GW, Rubin DB. Identification of causal effects using instrumental variables. J Am Stat Assoc. 1996;91(434):444–55. 10.1080/01621459.1996.10476902.

[22] Newhouse JP, McClellan M. Econometrics in outcomes research: the use of instrumental variables. Annu Rev Public Health. 1998;19:17–34. 10.1146/annurev.publhealth.19.1.17. 9611610 10.1146/annurev.publhealth.19.1.17

[23] Allen N, Sudlow C, Downey P, et al. UK Biobank: current status and what it means for epidemiology. Health Policy Technol. 2012;1(3):123–6. 10.1016/j.hlpt.2012.07.003.

[24] Davey Smith G, Ebrahim S. ‘Mendelian randomization’: Can genetic epidemiology contribute to understanding environmental determinants of disease? Int J Epidemiol. 2003;32(1):1–22. 10.1093/ije/dyg070. 12689998 10.1093/ije/dyg070

[25] Palmer TM, Lawlor DA, Harbord RM, et al. Using multiple genetic variants as instrumental variables for modifiable risk factors. Stat Methods Med Res. 2012;21(3):223–42. 10.1177/0962280210394459. 21216802 10.1177/0962280210394459 PMC3917707

[26] Pierce BL, Burgess S. Efficient design for Mendelian randomization studies: subsample and 2-sample instrumental variable estimators. Am J Epidemiol. 2013;178(7):1177–84. 10.1093/aje/kwt084. 23863760 10.1093/aje/kwt084 PMC3783091

[27] Millwood IY, Walters RG, Mei XW, et al. Conventional and genetic evidence on alcohol and vascular disease aetiology: a prospective study of 500 000 men and women in China. Lancet. 2019;393(10183):1831–42. 10.1016/S0140-6736(18)31772-0. 30955975 10.1016/S0140-6736(18)31772-0 PMC6497989

[28] Hamilton FW, Hughes DA, Spiller W, Tilling K, Davey SG. Non-linear Mendelian randomization: detection of biases using negative controls with a focus on BMI, Vitamin D and LDL cholesterol. Eur J Epidemiol. 2024. 10.1007/s10654-024-01113-9. 38789826 10.1007/s10654-024-01113-9 PMC11219394

[29] Power GM, Sanderson E, Pagoni P, et al. Methodological approaches, challenges, and opportunities in the application of Mendelian randomisation to lifecourse epidemiology: a systematic literature review. Eur J Epidemiol. 2024;39(5):501–20. 10.1007/s10654-023-01032-1. 37938447 10.1007/s10654-023-01032-1 PMC7616129

[30] Caspi A, Moffitt TE, Morgan J, et al. Maternal expressed emotion predicts children’s antisocial behavior problems: using monozygotic-twin differences to identify environmental effects on behavioral development. Dev Psychol. 2004;40(2):149–61. 10.1037/0012-1649.40.2.149. 14979757 10.1037/0012-1649.40.2.149

[31] D’Onofrio BM, Turkheimer EN, Eaves LJ, et al. The role of the children of twins design in elucidating causal relations between parent characteristics and child outcomes. J Child Psychol Psychiatry. 2003;44(8):1130–44. 10.1111/1469-7610.00196. 14626455 10.1111/1469-7610.00196

[32] Brion MJA, Lawlor DA, Matijasevich A, et al. What are the causal effects of breastfeeding on IQ, obesity and blood pressure? Evidence from comparing high-income with middle-income cohorts. Int J Epidemiol. 2011;40(3):670–80. 10.1093/ije/dyr020. 21349903 10.1093/ije/dyr020 PMC3147072

[33] Desai JR, Hyde CL, Kabadi S, et al. Utilization of positive and negative controls to examine comorbid associations in observational database studies. Med Care. 2017;55(3):244–51. 10.1097/MLR.0000000000000640. 27787351 10.1097/MLR.0000000000000640 PMC5318155

[34] Lu H, Cole SR, Howe CJ, Westreich D. Toward a clearer definition of selection bias when estimating causal effects. Epidemiol Camb Mass. 2022;33(5):699. 10.1097/EDE.0000000000001516. 10.1097/EDE.0000000000001516 PMC9378569 35700187

[35] Gleason CE, Norton D, Zuelsdorff M, et al. Association between enrollment factors and incident cognitive impairment in Blacks and Whites: data from the Alzheimer’s Disease Center. Alzheimers Dement J Alzheimers Assoc. 2019;15(12):1533. 10.1016/j.jalz.2019.07.015. 10.1016/j.jalz.2019.07.015 PMC6925619 31601516

[36] Davey Smith G, Lipsitch M, Tchetgen Tchetgen E, Cohen T. Negative control exposures in epidemiologic studies. Epidemiology. 2012;23(2):350–2. 22317815 10.1097/EDE.0b013e318245912c

[37] Howe LD, Matijasevich A, Tilling K, et al. Maternal smoking during pregnancy and offspring trajectories of height and adiposity: comparing maternal and paternal associations. Int J Epidemiol. 2012;41(3):722–32. 10.1093/ije/dys025. 22407859 10.1093/ije/dys025 PMC3396309

[38] Banack HR, Kaufman JS, Wactawski-Wende J, Troen BR, Stovitz SD. Investigating and remediating selection bias in geriatrics research: the selection bias toolkit. J Am Geriatr Soc. 2019;67(9):1970–6. 10.1111/jgs.16022. 31211407 10.1111/jgs.16022 PMC9930538

[39] Banack HR, Hayes-Larson E, Mayeda ER. Monte Carlo simulation approaches for quantitative bias analysis: a tutorial. Epidemiol Rev. 2021;43(1):106–17. 10.1093/epirev/mxab012. 10.1093/epirev/mxab012 PMC9005059 34664653

[40] Mayeda ER, Tchetgen Tchetgen EJ, Power MC, et al. A Simulation platform for quantifying survival bias: an application to research on determinants of cognitive decline. Am J Epidemiol. 2016;184(5):378–87. 10.1093/aje/kwv451. 27578690 10.1093/aje/kwv451 PMC5013884

[41] Tchetgen EJT, Walter S, Vansteelandt S, Martinussen T, Glymour M. Instrumental variable estimation in a survival context. Epidemiol Camb Mass. 2015;26(3):402. 10.1097/EDE.0000000000000262. 10.1097/EDE.0000000000000262 PMC4387894 25692223

[42] Althubaiti A. Information bias in health research: definition, pitfalls, and adjustment methods. J Multidiscip Healthc. 2016;9:211–7. 10.2147/JMDH.S104807. 27217764 10.2147/JMDH.S104807 PMC4862344

[43] Davey Smith G, Ebrahim S. Mendelian randomization: prospects, potentials, and limitations. Int J Epidemiol. 2004;33(1):30–42. 10.1093/ije/dyh132. 15075143 10.1093/ije/dyh132

[44] Morris TT, Heron J, Sanderson ECM, Davey Smith G, Didelez V, Tilling K. Interpretation of Mendelian randomization using a single measure of an exposure that varies over time. Int J Epidemiol. 2022;51(6):1899–909. 10.1093/ije/dyac136. 35848950 10.1093/ije/dyac136 PMC9749705

[45] Holmes MV, Davey Smith G. Revealing the effect of CETP inhibition in cardiovascular disease. Nat Rev Cardiol. 2017;14(11):635–6. 10.1038/nrcardio.2017.156. 28980665 10.1038/nrcardio.2017.156 PMC5644574

[46] Sobczyk MK, Zheng J, Davey Smith G, Gaunt TR. Systematic comparison of Mendelian randomisation studies and randomised controlled trials using electronic databases. BMJ Open. 2023;13(9): e072087. 10.1136/bmjopen-2023-072087. 37751957 10.1136/bmjopen-2023-072087 PMC10533809

[47] Rosenbaum PR. Replication and evidence factors in observational studies. 1st ed. CRC Press; 2021.

[48] Labrecque J, Swanson SA. Understanding the assumptions underlying instrumental variable analyses: a brief review of falsification strategies and related tools. Curr Epidemiol Rep. 2018;5(3):214–20. 10.1007/s40471-018-0152-1. 30148040 10.1007/s40471-018-0152-1 PMC6096851

[49] Shapland CY, Bell JA, Borges MC, et al. A quantitative approach to evidence triangulation: development of a framework to address rigour and relevance. Published online September 23, 2024:2024.09.20.24314046. 10.1101/2024.09.20.24314046

[50] Rehm J, Gmel GE, Gmel G, et al. The relationship between different dimensions of alcohol use and the burden of disease-an update. Addict Abingdon Engl. 2017;112(6):968–1001. 10.1111/add.13757. 10.1111/add.13757 PMC5434904 28220587

[51] Scherr PA, LaCroix AZ, Wallace RB, et al. Light to moderate alcohol consumption and mortality in the elderly. J Am Geriatr Soc. 1992;40(7):651–7. 10.1111/j.1532-5415.1992.tb01954.x. 1607579 10.1111/j.1532-5415.1992.tb01954.x

[52] Collins MA, Neafsey EJ, Mukamal KJ, et al. Alcohol in moderation, cardioprotection, and neuroprotection: epidemiological considerations and mechanistic studies. Alcohol Clin Exp Res. 2009;33(2):206–19. 10.1111/j.1530-0277.2008.00828.x. 19032583 10.1111/j.1530-0277.2008.00828.x PMC2908373

[53] Collins SE. Associations between socioeconomic factors and alcohol outcomes. Alcohol Res Curr Rev. 2016;38(1):83–94. 10.35946/arcr.v38.1.11 PMC4872618 27159815

[54] Xu W, Wang H, Wan Y, et al. Alcohol consumption and dementia risk: a dose-response meta-analysis of prospective studies. Eur J Epidemiol. 2017;32(1):31–42. 10.1007/s10654-017-0225-3. 28097521 10.1007/s10654-017-0225-3

[55] Handing EP, Andel R, Kadlecova P, Gatz M, Pedersen NL. Midlife alcohol consumption and risk of dementia over 43 years of follow-up: a population-based study from the Swedish twin registry. J Gerontol A Biol Sci Med Sci. 2015;70(10):1248–54. 10.1093/gerona/glv038. 25881581 10.1093/gerona/glv038

[56] Järvenpää T, Rinne JO, Koskenvuo M, Räihä I, Kaprio J. Binge drinking in midlife and dementia risk. Epidemiol Camb Mass. 2005;16(6):766–71. 10.1097/01.ede.0000181307.30826.6c. 10.1097/01.ede.0000181307.30826.6c 16222166

[57] Andrews SJ, Goate A, Anstey KJ. Association between alcohol consumption and Alzheimer’s disease: a Mendelian randomization Study. Alzheimers Dement J Alzheimers Assoc. 2020;16(2):345–53. 10.1016/j.jalz.2019.09.086. 10.1016/j.jalz.2019.09.086 PMC7057166 31786126

[58] Zheng L, Liao W, Luo S, et al. Association between alcohol consumption and incidence of dementia in current drinkers: linear and non-linear mendelian randomization analysis. eClinicalMedicine. 2024. 10.1016/j.eclinm.2024.102810. 39290634 10.1016/j.eclinm.2024.102810 PMC11405827

[59] Tierney BT, Anderson E, Tan Y, et al. Leveraging vibration of effects analysis for robust discovery in observational biomedical data science. PLOS Biol. 2021;19(9): e3001398. 10.1371/journal.pbio.3001398. 34555021 10.1371/journal.pbio.3001398 PMC8510627

[60] Loder E, Groves T, Macauley D. Registration of observational studies. BMJ. 2010;340: c950. 10.1136/bmj.c950. 20167643 10.1136/bmj.c950

[61] Lash TL, Vandenbroucke JP. Should preregistration of epidemiologic study protocols become compulsory? Reflections and a counterproposal. Epidemiology. 2012;23(2):184–8. 10.1097/EDE.0b013e318245c05b. 22317802 10.1097/EDE.0b013e318245c05b

[62] Griffith LE, van den Heuvel E, Fortier I, et al. Statistical approaches to harmonize data on cognitive measures in systematic reviews are rarely reported. J Clin Epidemiol. 2015;68(2):154–62. 10.1016/j.jclinepi.2014.09.003. 25497980 10.1016/j.jclinepi.2014.09.003 PMC4685455

[63] Chen D, Jutkowitz E, Iosepovici SL, Lin JC, Gross AL. Pre-statistical harmonization of behavioral instruments across eight surveys and trials. BMC Med Res Methodol. 2021;21(1):227. 10.1186/s12874-021-01431-6. 34689753 10.1186/s12874-021-01431-6 PMC8543796

[64] Davey Smith G Increasing the accessibility of data. BMJ. 1994;308(6943):1519–20. 10.1136/bmj.308.6943.1519. 8019302 10.1136/bmj.308.6943.1519 PMC2540522

[65] Glymour MM, Vittinghoff E. Commentary: selection bias as an explanation for the obesity paradox: just because it’s possible doesn’t mean it’s plausible. Epidemiology. 2014;25(1):4–6. 10.1097/EDE.0000000000000013. 24296924 10.1097/EDE.0000000000000013

[66] Greenland S. Quantifying biases in causal models: classical confounding vs collider-stratification bias. Epidemiology. 2003;14(3):300–6. 12859030

[67] Davey Smith G, Phillips AN. Correlation without a cause: an epidemiological odyssey. Int J Epidemiol. 2020;49(1):4–14. 10.1093/ije/dyaa016. 32244255 10.1093/ije/dyaa016

[68] Claveau F. The independence condition in the variety-of-evidence thesis. Philos Sci. 2013;80(1):94–118. 10.1086/668877.

[69] Osimani B, Landes J. Varieties of error and varieties of evidence in scientific inference. Br J Philos Sci. 2023;74(1):117–70. 10.1086/714803.

[70] Bateson W. The Methods and Scope of Genetics: An Inaugural Lecture delivered 23 October 1908. Published online http://www.esp.org/foundations/genetics/classical/holdings/b/wb-methods-08.pdf

[71] Hill AB. The environment and disease: Association or causation? Proc R Soc Med. 1965;58(5):295–300. 14283879 10.1177/003591576505800503 PMC1898525

[72] Clayton A. Bernoulli’s Fallacy: Statistical Illogic and the Crisis of Modern Science. Columbia University Press, USA. 2021

